# Specifically regulated genes in malignant melanoma tissues identified by subtractive hybridization

**DOI:** 10.1054/bjoc.1999.1055

**Published:** 2000-02-21

**Authors:** R Hipfel, B Schittek, Y Bodingbauer, C Garbe

**Affiliations:** Section of Dermatologic Oncology, Department of Dermatology, Eberhard-Karls-University Tuebingen, Liebermeisterstr. 25, Tuebingen, 72076, Germany

**Keywords:** subtractive hybridization, array hybridization, differential gene expression, malignant melanoma, melanocyticnaevi

## Abstract

A polymerase chain reaction (PCR)-based subtractive hybridization technique was used to identify transformation-related genes in malignant melanoma. Melanoma biopsies were compared with tissues of benign melanocytic naevi and 549 gene fragments were screened using arrayed filters. Thirty-eight clones were confirmed to be differentially expressed representing 30 different genes (18 melanoma-specific and 12 naevus-specific genes). To further confirm differential gene expression, Northern blot analyses with six of the 30 genes as probes were performed. All six were differentially expressed in benign and malignant melanocytic lesions, specifically dbpB/YB-1, 67-kDa laminin receptor, CAGH-3, 71-kDa heat shock protein and two unknown genes. The expression levels of these genes were then analysed in 50 different tissues to determine their overall expression profile. In conclusion, the technique of PCR-based subtractive hybridization in combination with arrayed filters allows detection of differences in gene expression even in tissues from which high-quality RNA is hard to isolate. The genes identified in this study are of interest because of their potential role in the pathogenesis of malignant melanoma. © 2000 Cancer Research Campaign


*British Journal of Cancer* (2000) **82**(6), 1149–1157
© 2000 Cancer Research Campaign
Article no. bjoc.1999.1055, available online at http://www.idealibrary.com on IDEAL®

### Specifically regulated genes in malignant melanoma tissues identified by subtractive hybridization

**R Hipfel, B Schittek, Y Bodingbauer and C Garbe**

Section of Dermatologic Oncology, Department of Dermatology, Eberhard-Karls-University Tuebingen, Liebermeisterstr. 25, 72076 Tuebingen, Germany

**Summary** A polymerase chain reaction (PCR)-based subtractive hybridization technique was used to identify transformation-related genes in malignant melanoma. Melanoma biopsies were compared with tissues of benign melanocytic naevi and 549 gene fragments were screened using arrayed filters. Thirty-eight clones were confirmed to be differentially expressed representing 30 different genes (18 melanoma-specific and 12 naevus-specific genes). To further confirm differential gene expression, Northern blot analyses with six of the 30 genes as probes were performed. All six were differentially expressed in benign and malignant melanocytic lesions, specifically dbpB/YB-1, 67-kDa laminin receptor, CAGH-3, 71-kDa heat shock protein and two unknown genes. The expression levels of these genes were then analysed in 50 different tissues to determine their overall expression profile. In conclusion, the technique of PCR-based subtractive hybridization in combination with arrayed filters allows detection of differences in gene expression even in tissues from which high-quality RNA is hard to isolate. The genes identified in this study are of interest because of their potential role in the pathogenesis of malignant melanoma. © 2000 Cancer Research Campaign

**Keywords:** subtractive hybridization; array hybridization; differential gene expression; malignant melanoma; melanocytic naevi

Melanocytic naevi, dysplastic naevi, horizontally growing melanoma (radial growth phase), invasively growing melanoma (vertical growth phase), and metastasizing melanoma are thought to represent successive stages in the tumour progression of the melanocytic lineage. Melanocytic transformation is accompanied by specific changes in gene expression. Some 200–300 genes are supposed to be differentially expressed between normal and cancer cells (Zhang et al, 1997) contributing to the malignant phenotype of tumours. Several genes have already been identified to be differentially expressed in malignant melanoma. Specific examples include the melanoma-associated antigens MAGE and PRAME (van der Bruggen et al, 1991; Ikeda et al, 1997), the tumour suppressor p16/CDKN2 (Reed et al, 1995), the melanocyte lineage marker Pmel17/gp100 (Wagner et al, 1997), and the epidermal growth factor (EGF) receptor (Real et al, 1986). Nevertheless, further data is needed to provide better insight in the tumour biology of melanoma because individually none of these genes has been shown to be aetiologically involved in tumour progression (Herlyn, 1993). It is also well accepted that multiple and independent events lead to an accumulation of genetic defects that determine the malignant phenotype of tumours.

Gene expression patterns in vitro also differ from those in vivo because of the cell culture conditions and the altered microenvironment of the cells. Results obtained from tumour tissues may therefore be more reliable than those from cell lines. However, when dealing with tissues there are significant problems such as the limited number of primary tumour samples available, the heterogeneity of the tissues, individual tissue variations, and the difficulty in isolating high-quality RNA in sufficient amounts from biopsies that are hard to homogenize (e.g. skin). We therefore used the following strategies to overcome these problems: (i) A modified RNA isolation step was established to obtain high-quality RNA from skin biopsies (Hipfel et al, 1998); (ii) for difference analysis five primary melanoma and five melanocytic naevi were pooled, respectively, to reduce the number of genes obtained due to individual tissue variations; (iii) a polymerase chain reaction (PCR)-based subtractive hybridization method was applied that allows the enrichment of low abundant differential transcripts and requires less RNA than conventional subtractive hybridization techniques; (iv) arrayed filters were used for high-throughput gene expression difference analysis (reverse Northern blot); and (v) finally Northern blot analysis using selected gene fragments as probes were used to confirm differential gene expression in a range of relevant tissues and cell lines. This procedure led to the identification of several genes, including some unknown genes and expressed sequence tags, that have never previously been associated with melanocytic transformation.

*Received 7 April 1999*
*Revised 27 August 1999*
*Accepted 23 September 1999*

*Correspondence to:* C Garbe

### MATERIALS AND METHODS

### Tissue specimen collection and RNA extraction

A total of 100–250 mg of five benign congenital melanocytic naevi (measuring less than 10 cm in diameter) and five primary melanoma tissues (tumour thickness ranged between 1.04 mm and 3.3 mm) were snap-frozen in liquid nitrogen immediately after resection. Benign congenital instead of acquired melanocytic naevi were used to get sufficient RNA for the subtraction. None of these congenital melanocytic naevi demonstrated any signs of architectural disorder or cytologic atypia in the naevocytes. Tissue samples were also collected from skin (safety margin of melanoma surgical specimen) and melanoma metastases of the skin and lymph nodes. High-quality total RNA was isolated using our modified procedure based on a guanidinium thiocyanate (GTC)–phenol mixture (RNA-Clean™; AGS, Heidelberg, Germany) as described previously (Hipfel et al, 1998). Integrity of the RNA was checked by gel electrophoresis revealing two bright bands corresponding to ribosomal 28S and 18S RNA at about 4.5 and 1.9 kb, respectively, with a ratio not smaller than 1:1. Poly-adenosine (Poly-A) RNA was isolated from the total RNA preparation using the Quickprep Micro mRNA Purification Kit (Pharmacia Biotech, Freiburg, Germany) with yields between 1 and 5%.

### PCR-based subtractive hybridization

Reciprocal subtractive hybridizations were carried out between cDNAs from melanoma and naevi tissues using the PCR-Select™ cDNA Subtraction Kit (Clontech, Palo Alto, CA, USA) as described in the instructions to enrich for both melanoma-specific and naevus-specific transcripts.

Briefly, poly-A RNA from five melanoma biopsies and five congenital melanocytic naevi from different patients were pooled respectively. As starting material, 2 μg poly-A RNA each was used for first-strand and second-strand cDNA synthesis. After *Rsa*I-digestion and adaptor ligation hybridization of tester and driver were performed for 8 h (first hybridization) and 15 h (second hybridization) at 68°C. Two PCR steps were performed to amplify differentially expressed genes (first PCR: 27 cycles of 94°C 30 s, 66°C 30 s and 72°C 1.5 min; nested PCR: 12 cycles of 94°C 30 s, 66°C 30 s and 72°C 1.5 min) using the 50 × Advantage KlenTaq Polymerase Mix (Clontech). Efficiencies of *Rsa*I-digestions, adaptor ligations and subtractive hybridizations were checked as recommended in the kit.

Subtracted cDNAs were inserted into the *Eco*RV site of pBluescript® II SK (Stratagene, Heidelberg, Germany) and transformed into INVαF′ cells (Invitrogen, Leek, The Netherlands).

### Arrayed filters and hybridization analysis (reverse Northern blot)

To isolate individual cDNAs of the subtracted library single bacterial transformants were incubated in 100 μl LB amp (50 μg $ml^{-1}$) at 37°C for at least 4 h. Inserts were PCR amplified (95°C 30 s, 56°C 30 s and 72°C 2.5 min for 30 cycles) in 20 μl containing 10 mM Tris–HCl pH 9.0, 1.5 mM magnesium chloride ($MgCl_2$), 50 mM potassium chloride (KCl), 200 μM dNTP, 0.5 μM vector-specific SK and KS primers, 1.5 Units *Taq* polymerase (Pharmacia Biotech), and 1 μl of bacterial culture.

Then, 1.5 μl of a mixture containing 3 μl PCR-amplified inserts and 2 μl 0.3 N sodium hydroxide (NaOH)/15% Ficoll were spotted onto a 10 × 10 cm positively charged nylon membrane (Boehringer Mannheim, Germany). In this way, 100 spots were arrayed on duplicate filters for subsequent hybridization. The first differential screening step consists of hybridizations of the subtracted library with itself to minimize background (Wang and Brown, 1991). The probes were made of the nested PCR product of the subtractions with melanoma as tester (melanoma subtracted probe) or naevi as tester (naevi subtracted probe) following the instructions of the Clontech subtraction kit. Labelling with Digoxigenin was performed with the DIG DNA Labelling Kit (Boehringer Mannheim). Hybridizations were carried out overnight in a buffer containing 6 × SSC (saline–sodium citrate), 50 μg $ml^{-1}$ denatured salmon sperm DNA, 5 × Denhardt's solution (0.2% (w/v) Ficoll 400, polyvinylpyrrolidone and bovine serum albumin (BSA) each), and 0.5% sodium dodecyl sulphate (SDS) at 65°C. The filters were washed twice in 2 × SSC/0.5% SDS at 65°C for 10 min and twice in 1 × SSC/0.5% SDS at 65°C for 10 min, and subjected to detection using anti-DIG–AP conjugates and CDP-Star™ as chemiluminescent substrate according to the instructions of the DIG DNA Detection Kit (Boehringer Mannheim). Blots were exposed to Kodak X-OMAT chemiluminescent film at room temperature for several minutes.

Clones confirmed to be differentially expressed in this first screening step were again arrayed on duplicate filters as described above and subjected to the second differential screening step with hybridizing probes made of unsubtracted cDNA from melanoma tissues (melanoma cDNA probe) and unsubtracted cDNA from naevus tissues (naevi cDNA probe). RNA used for the synthesis of these probes were from the same pool of RNA used for subtraction. Probes were labelled with Digoxigenin during cDNA-synthesis in 40 μl containing 1 μg mRNA, 50 mM Tris–HCl pH 8.3, 75 mM KCl, 3 mM $MgCl_2$, 2.5 μM dithiothreitol (DTT), 4 μl 10 × DIG dNTP labelling mixture (Boehringer Mannheim), 1 μM T20-primer, and 100 Units SuperScript™ II RNAase $H^-$ reverse transcriptase (Gibco-BRL, Eggenstein, Germany) for 1 h at 42°C. Hybridization, washing of the filters, and detection of the signals were performed exactly as described for the first screening step.

### Sequencing and computer analysis

Cloned cDNAs that were confirmed to be differentially expressed in the first and second differential screening step were sequenced using the BigDye Terminator Cycle Sequencing reagents (Perkin-Elmer, Foster City, CA, USA) and an ABI373 DNA Sequencer. Identification of sequences was performed using the non-redundant database of GenBank, EMBL, DDBJ and PDB (BLAST program).

### Northern analysis

To further confirm differential gene expression 1 μg mRNA from several melanoma, melanocytic naevi, skin, melanoma metastases and melanoma cell lines was fractionated on a 1% Northern gel and transferred to a positively charged nylon membrane (Boehringer Mannheim). The blot was hybridized with DIG-labelled cDNA fragments of the confirmed clones (67-kDa laminin receptor, dbpB/YB-1, CAGH-3, 71-kDa heat shock protein, novel gene, EST ac73 g10.sl). Hybridization was performed overnight at 50°C in DIG Easy Hyb (Boehringer Mannheim). The filters were washed twice in 2 × SSC / 0.1% SDS at room temperature for 2 min and twice in 2 × SSC / 0.1% SDS at 50°C for 15 min, and subjected to Digoxigenin detection as described above. Hybridization signals were quantified by scanning the chemiluminescent film and volume integration using the Molecular Analyst™ program (Bio-Rad Laboratories, Hercules, CA, USA).

Expression patterns of differentially expressed genes in other human tissues were determined using the RNA Master Blot™ (Clontech) with dots of poly-A RNA from 50 different tissues and developmental stages. Hybridizing probes were radioactive labelled with the Klenow fragment of DNA polymerase I (MBI

*© 2000 Cancer Research Campaign*

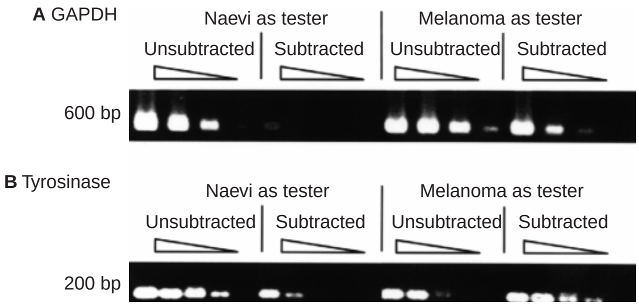

**Figure 1** PCR analysis of subtraction efficiency. After reciprocal subtractive hybridizations with melanoma and naevi as tester, respectively, GAPDH **(A)** and tyrosinase **(B)** were PCR-amplified with 18, 23, 28 and 33 cycles in **(A)**, and 10, 15, 20, 25 and 30 cycles in **(B)** indicated by triangles with subtracted and unsubtracted cDNA. This shows that the abundant housekeeping gene GAPDH is significantly reduced in both subtractions, and tyrosinase is enriched in the subtraction with melanoma as tester and reduced in the subtraction with naevi as tester

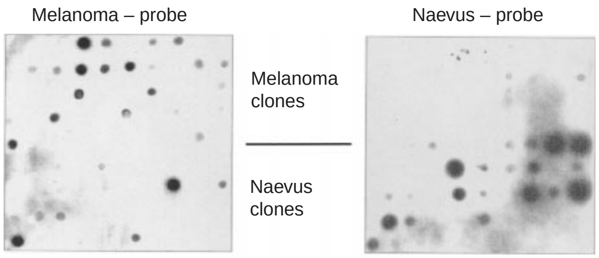

**Figure 2** First differential screening step using arrayed filters hybridized with subtracted cDNA probes. Individual clones of the subtracted libraries were spotted onto duplicate nylon membranes (clones of the subtraction with melanoma as tester are localized in the upper halves of the filters, clones of the subtractions with naevi as tester in the lower halves). The arrayed filters were hybridized with a melanoma subtracted probe (left) and a naevi subtracted probe (right) revealing most genes to be differentially expressed and some to be false-positive or false-negative

**Table 1** Array hybridization with unsubtracted cDNA as probes

| | Number of clones | Non-differential clones (%) | Clones without detectable signal (%) | Differentially expressed clones (%) |
|---|---|---|---|---|
| Melanoma as tester | 174 | 19 (11) | 133 (76) | 22 (13) |
| Naevi as tester | 163 | 39 (24) | 108 (66) | 16 (10) |
| Total | 337 | 58 (17) | 241 (72) | 38 (11) |

Fermentas) in the presence of 50 μCi [α-$^{32}$P]dCTP (Amersham) and 1 μg hexamer primer (Feinberg and Vogelstein, 1983). Hybridization and washing were carried out according to the manufacturers' instructions. Filters were exposed to an autoradiography film, and hybridization signals were quantified using a Phosphorimager (Fujifilm BAS 1500).

### RESULTS

### PCR-based subtractive hybridization comparison of gene expression patterns between malignant melanoma and melanocytic naevus tissues

Reciprocal PCR-based subtractive hybridizations were carried out between primary melanoma and melanocytic naevi cDNAs to identify melanoma-specific genes (melanoma as tester) and naevus-specific genes (naevi as tester). Subtraction efficiency was determined using a semi-quantitative PCR for gluceraldehyde 3-phosphate dehydrogenase (GAPDH). A significant reduction of the cDNA of the abundant housekeeping gene GAPDH was observed in subtracted compared to unsubtracted cDNA for both naevi as tester and melanoma as tester indicating that the subtractive hybridizations were successful (Figure 1A). Differences of about seven cycles and 13 cycles for equal amplification of GAPDH were seen in the subtraction with melanoma as tester and with naevi as tester, respectively, suggesting that the latter was more efficient (five cycles correspond roughly to a 20-fold cDNA concentration difference).

For further verification of successful subtractive hybridizations tyrosinase, a melanocytic lineage marker known to be overexpressed in melanoma compared to naevi (Herlyn, 1993) was amplified. As seen in Figure 1B, an enrichment of tyrosinase transcripts was seen in the subtraction with melanoma as tester and a reduction of tyrosinase transcripts with naevi as tester.

Moreover, the melanoma-associated antigen PRAME (Ikeda et al, 1997) was present in much higher quantities in the melanoma-specific subtracted library than in the naevus specific subtracted library (data not shown).

### Use of arrayed filters to screen large numbers of cDNA inserts of the subtracted library

The enriched cDNAs of the subtractions were cloned, and individual inserts were PCR amplified and spotted onto duplicate nylon membranes (arrayed filters). Insert sizes ranged between 200 bp and 1400 bp (average size was about 400 bp). In the first differential screening step the clones were hybridized with a probe made of the subtracted library itself (Wang et al, 1991) to eliminate non-differential clones (Figure 2). Of 549 clones, 337 (61%) revealed differential signals (174 melanoma-specific and 163 naevus-specific clones) and were therefore subjected to the second differential hybridization step with probes made of unsubtracted cDNA from melanoma tissues and naevus tissues, respectively. A high percentage of the clones (72%) revealed no signal at all, suggesting that among these cDNA low abundant transcripts are present that cannot be detected in this way. Thirty-eight clones (11%) were confirmed to be differentially expressed, 22 melanoma-specific clones and 16 naevus-specific clones (Table 1). These clones were sequenced and analysed by database searching with the BlastN program (Altschul et al, 1997). In Figure 3 representative clones of the arrayed filters are

© 2000 Cancer Research Campaign

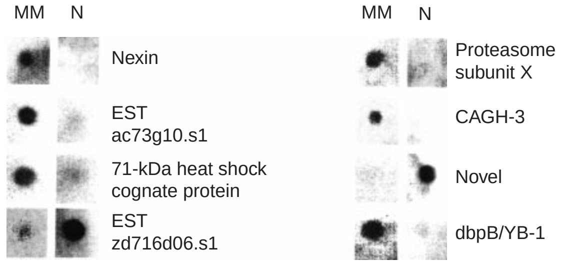

**Figure 3** Second differential screening step using arrayed filters hybridized with unsubtracted cDNA probes. The clones confirmed in the first screening step were again spotted onto duplicate filters and hybridized with unsubtracted cDNA from melanoma (MM) and unsubtracted cDNA from naevi (N) respectively. Eight of the 38 clones that were confirmed in both screening steps are shown (six melanoma-specific genes and two naevus-specific genes)

using RNA from different sources: skin, common congenital melanocytic naevi, primary melanoma, lymph node and skin metastases of melanoma as well as various melanoma cell lines. Quantification of the hybridization signals revealed all six genes to be differentially expressed between benign and malignant melanocytic lesions (Figure 4). Interestingly, expression of one gene, the 67-kDa laminin receptor, showed a correlation with tumour progression (Figure 4A) that is consistent with the data in the literature (Vacca et al, 1993) but contradicts the result of the arrayed hybridization where the gene is preferentially expressed in naevi (Table 3).

Expression of *dbpB/YB-1* correlates with tumour progression since skin and three of three naevi showed almost no signal, whereas all of the examined melanoma, melanoma metastases and melanoma cell lines revealed significant stronger signals (Figures 4B and 5A).

**Table 2** Gene fragments expressed preferentially in malignant melanoma (reverse Northern)

| Clone | Accession number | Identity | Approximate insert size | Homology region |
|---|---|---|---|---|
| 1 | S73003 | gp100 | 200 | 1900–2011 |
| 2 | U03698 | HLA B-40011 | 300 | 856–560 |
| 3 | M87790 | Ig λ anti-hepatitis A | 400 | 588–198 |
| 4 | M21731 | Lipocortin V | 550 | 778–1285 |
| 5 | X58079 | S-100 | 210 | 194–59 |
| 6 | U80747 | CAGH-3 | 520 | 2001–1694 |
| 7 | M17783 | Glia-derived nexin | 600 | 120–583 |
| 8 | X04106 | Calpain | 500 | 657–1100 |
| 9 | S73003 | gp100 | 350 | 1903–1588 |
| 10 | S73003 | gp100 | 180 | 1181–1021 |
| 11 | L28809 | dbpB/YB-1 | 650 | 925–578 |
| 12 | M95708 | CD59 | 350 | 325–575 |
| 13 | D29011 | Proteasome subunit X | 220 | 911–1018 |
| 14 | M33519 | BAT3 | 800 | 3025–2648 |
| 15 | S73003 | gp100 | 550 | 652–852 |
| 16 | AC002431 | Human BAC clone RG180F08 | 700 | 106029–106280 |
| 17 | T59964 | EST yb67e03.r1 | 360 | 161–5 |
| 18 | X15183 | 90-kDa heat shock protein | 700 | 2293–2110 |
| 19 | AA470116 | EST zt98g04.r1 | 600 | 437–312 |
| 20 | AA633960 | EST ac73g10.s1 | 700 | 285–656 |
| 21 | S73003 | gp100 | 600 | 481–905 |
| 22 | Y00371 | 71-kDa heat shock cognate protein | 600 | 1032–1244 |

Gene fragments preferentially expressed in primary malignant melanoma identified by arrayed filter hybridizations. Bold lettering indicates the genes that were analysed on Northern blot. The homology search was performed using the BLAST program with sequence identities between 93% and 100%. The homology region corresponds to the nucleotide positions within the GenBank sequence.

shown, and in Tables 2 and 3 all clones are listed with the insert sizes and the GenBank matches. Among these, there were several genes already known to be differentially expressed (e.g. S-100, gp 100), some genes that have never been associated with the malignant phenotype (e.g. CAGH-3, dbpB/YB-1), and some unknown sequences (novel genes and expressed sequence tags).

### Northern analyses to examine expression of genes in a range of tissues and cell lines

To further confirm differential gene expression between benign and malignant melanocytic lesions, and to investigate the expression profile during melanoma progression, six genes were selected (marked in Tables 2 and 3) to use as probes on Northern blots

*CAGH-3* was not found in skin and in none of the eight naevi examined, whereas a weak signal could be detected in one of five primary melanomas. A strong signal was seen in two of four lymph node metastases and in one of one skin metastasis. In addition, four of five melanoma cell lines were positive for CAGH-3 (Figure 4C).

The *novel gene* (clone 8 in Table 3) was strongly expressed in skin and in four of five melanocytic naevi but not in primary melanoma, lymph node metastases, skin metastasis and melanoma cell lines (Figures 4D and 5B). The transcript is about 0.5 kb in length and shows no homology to any known sequence (BLAST search and search in the TIGR database).

The *EST ac73 g10.sl* was not detected in skin, naevi and primary melanoma but was expressed in one of three lymph node

 © 2000 Cancer Research Campaign

**Table 3** Gene fragments expressed preferentially in melanocytic naevi (reverse Northern)

| Clone | Accession number | Identity | Approximate insert size | Homology region |
|---|---|---|---|---|
| 1 | M99061 | Epidermal cytokeratin 2 | 900 | 2040–1619 |
| 2 | M10938 | Epidermal 67-kDa type II keratin | 150 | 1848–1733 |
| 3 | M99061 | Epidermal cytokeratin 2 | 600 | 2046–1604 |
| 4 | M10938 | Epidermal 67-kDa type II keratin | 510 | 166–608 |
| 5 | M61120 | Loricrin | 200 | 1167–994 |
| 6 | W56586 | EST zd16d06.s1 | 1200 | 411–78 |
| 7 | J03464 | Collagen alpha-2 type I | 900 | 5338–4933 |
| 8 | | Novel | 400 | |
| 9 | X89401 | Large subunit of ribosomal protein L21 | 300 | 382–149 |
| 10 | M17733 | Thymosin beta-4 | 500 | 249–547 |
| 11 | M54927 | Myelin proteolipid protein | 280 | 1295–1137 |
| 12 | S37431 | 67-kDa laminin receptor | 700 | 125–555 |
| 13 | M10938 | Epidermal 67-kDa type II keratin | 280 | 1848–1917 |
| 14 | AF052153 | Aspartate aminotransferase | 150 | 1517–1344 |
| 15 | X06537 | Lecithin-Cholesterol acyltransferase | 400 | 1–158 |
| 16 | | Novel | 500 | |

Gene fragments preferentially expressed in melanocytic naevi identified by arrayed filter hybridizations. Bold lettering indicates the genes that were analysed on Northern blot. The homology search was performed using the BLAST program with sequence identities between 93% and 100%. The homology region corresponds to the nucleotide positions within the GenBank sequence. Clones that show no homology to any known sequence (non-redundant database of GenBank, EMBL, DDBJ and PDB, as well as GenBank EST division) are denoted as novel.

metastases, in one of one skin metastasis, and in five of five melanoma cell lines. This suggests that this gene is up-regulated in malignant melanocytes (Figure 4E). The transcript is about 2.9 kb in length and shows homology to the 3’end of murine necdin (367 of 372 bases).

Finally, the *71-kDa heat shock cognate protein* is weakly expressed in skin and in four of four melanocytic naevi and is highly expressed in all melanoma cell lines, lymph node and skin metastases examined.

In addition, the RNA Master Blot™ (Clontech) with RNA from 50 different tissues and developmental stages was applied to determine the overall expression profiles of the novel gene, the EST ac73 g10.sl, CAGH-3 and dbpB/YB-1 that were confirmed to be differentially expressed in melanoma. The Master Blot was normalized to the mRNA expression levels of eight different housekeeping genes: ribosomal protein S9, 23-kDa highly basic protein, tubulin, phospholipase, HPRT, β-actin, G3PDH and ubiquitin. A probe for ubiquitin was used as control showing a fairly consistent hybridization signal from all sample dots (data not shown). The Master Blot also contained yeast total RNA and tRNA, *Escherichia coli* rRNA and DNA, as well as human *Cot*1 DNA as negative controls that all showed no detectable signal in any of the hybridizations. Hybridization with EST ac73 g10.sl as a probe revealed that the gene is mainly expressed in pituitary gland and in placenta. The novel gene (clone 8 in Table 3) showed no detectable signal in any of the 50 tissues analysed and seems therefore be expressed exclusively in melanocytic naevi and normal skin. dbpB/YB-1 is expressed in most tissues but mainly in skeletal muscle, testis, heart, fetal liver and fetal heart. Finally, CAGH-3 is most prominently expressed in placenta and to a much lower extent in ovary and brain.

### DISCUSSION

The identification of genes specifically overexpressed or repressed in tumour tissues is a key step towards the understanding of the malignant transformation process. In this study a PCR-based subtractive hybridization method was applied to detect genes differentially expressed in malignant and benign melanocytic lesions using primary melanoma and benign melanocytic naevus tissues. We obtained two subtracted cDNA libraries of good quality as demonstrated by a significant reduction of cDNA of the abundant housekeeping gene GAPDH in subtracted compared to unsubtracted cDNAs. Furthermore, the melanoma-associated antigen PRAME (Ikeda et al, 1997) was present in much higher quantities in the melanoma-specific library than in the naevus-specific library.

High-quality RNA is needed for this technique but this is difficult to extract from small amounts of skin tissues. We therefore established an optimized protocol as described previously (Hipfel et al, 1998). Only a few studies have so far used tissues as starting material, most studies comparing gene expression between cell lines. Analysis of genes in tissues, however, has two major advantages. First, gene expression patterns are not altered due to growth factors and other additions to the culture media, and second, genes can be identified that are differentially regulated in benign and malignant lesions due to cell–cell interactions between melanocytes, fibroblasts and keratinocytes. It is known, for example, that the gene expression profile of melanocytes is partly regulated by keratinocytes, e.g. the expression of the MUC18 adhesion receptor (Shih et al, 1994), and that escape from keratinocyte control is a first step towards melanocytic transformation. In addition, melanoma cells produce a large number of

*© 2000 Cancer Research Campaign*

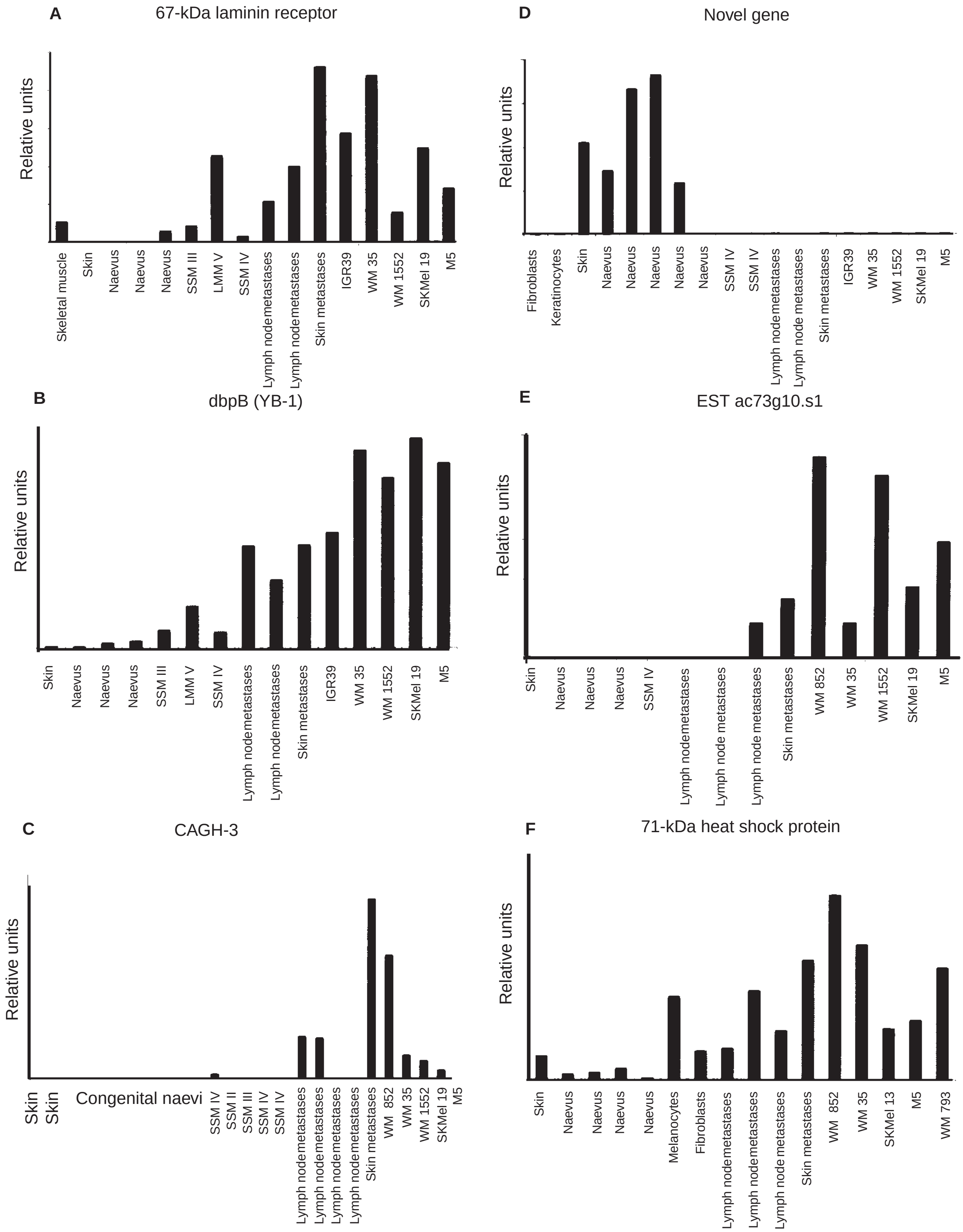

**Figure 4** Quantification of Northern hybridization signals of six genes by scanning and volume integration. The cloned transcript fragments of the 67-kDa laminin receptor **(A)**, the dbpB/YB-1 **(B)**, the CAGH-3 **(C)**, the novel gene **(D)**, the EST ac73 g10.s1 **(E)** and the 71-kDa heat shock protein **(F)** were used as probes on Northern blots. All genes were confirmed to be preferentially expressed in either malignant lesions **(A–C, E–F)** or in normal skin and benign lesions **(D)**. Depths of invasion of the primary melanoma (Clark level I–V) are indicated (Clark et al, 1969). 28S rRNA was used as control. Approximate sizes of the transcripts were 1 kb **(A)**, 1.6 kb **(B)**, 2.3 kb **(C)**, 0.5 kb **(D)**, 2.9 kb **(E)** and 2.3 kb **(F)**. Relative units (x-fold): Integrated volume of the bands on the Northern blot adjusted for background removal using the Molecular Analyst® Software (Bio-Rad)

 *© 2000 Cancer Research Campaign*

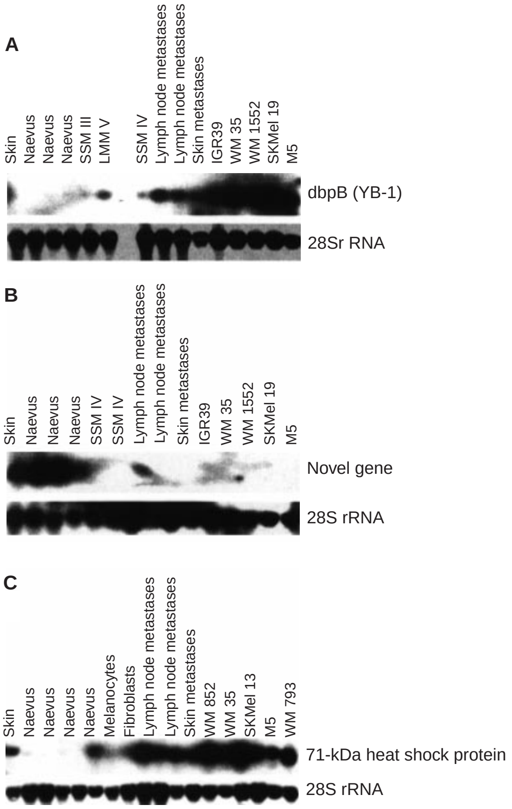

**Figure 5** Northern blot hybridizations. The cloned fragments of the dbpB/YB-1 **(A)**, the novel gene **(B)**, and the 71-kDa heat shock protein **(C)** were used as probes on Northern blots. The dbpB/YB-1 (1.6 kb) was confirmed to be overexpressed in melanoma, melanoma metastases, and melanoma cell lines, whereas the novel gene (0.5 kb) was confirmed to be preferentially expressed in benign lesions (naevi) and skin. The 71-kDa heat shock protein (2.3 kb) was preferentially expressed in malign melanocytic lesions. Although the product is almost not expressed in benign melanocytes in vivo (naevi), it is strongly expressed in melanocytes in vitro. Depths of invasion of the primary melanoma (Clark level I–V) are indicated (Clark et al, 1969)

soluble factors affecting the expression profile of the neighbouring cells which may also be of importance for the progression of the tumour (Herlyn and Shih, 1994). We therefore did not apply microdissection techniques to obtain all cell types of the skin for the subtraction analysis.

We demonstrated that PCR-based subtractive hybridization with RNA isolated from skin tissues is a powerful technique to identify a large number of differentially expressed genes. The method prevents amplification of common sequences and allows the enrichment of rare messages by means of the suppression PCR (Siebert et al, 1995; Diatchenko et al, 1996). The application of two consecutive differential screening procedures allowed a step-to-step elimination of non-differential clones. The first screening consisting of self-hybridizations of the subtracted library (Wang and Brown, 1991) led to the elimination of 212 of 549 analysed clones (39%) and is therefore a valuable tool to reduce the number of non-differential clones. The second screening consisting of hybridizations with unsubtracted cDNA as probes led to the elimination of another 58 non-differential clones (17%). The majority of clones, however, represented transcripts without detectable signals on the arrayed filters (Table 1). This is probably due to the low-abundance of these transcripts as they were detected with the amplified subtracted cDNA probe in the first screening step but not detected with the non-amplified unsubtracted cDNA probe in the second screening step. This high number of rare transcripts reflects the strength of the suppression PCR and it would be interesting to analyse with a different screening procedure how many of these rare transcripts are differentially expressed.

Sequencing of the 38 confirmed cDNA clones showed that a high number of different genes (30 of 38) were detected, reflecting the high quality of the subtracted library (Tables 2 and 3). Only gp100 and keratin was detected several times (five clones each). gp100 is a melanocytic lineage marker that is known to be significantly overexpressed in melanoma (Wagner et al, 1997). This result shows the high efficiency and reliability of the subtraction technique. S100, a calcium-binding protein that is diagnostic of a lesion of melanocytic origin, was found once in the melanoma-specific library and might as well be overexpressed in melanoma cells. In the naevus-specific library, two of the 16 identified genes are known to be expressed mainly in keratinocytes and were isolated several times (keratin and loricrin). The fact that many keratinocyte-specific genes are found in the naevus-specific library suggests that keratinocyte RNA is overrepresented in naevus tissues compared to melanoma biopsies. In the tumour tissues most likely the transcription of many different genes are switched on to facilitate the uncontrolled growth of the tumour cells. This can result in the underrepresentation of keratinocyte-specific genes in the melanoma-specific library.

Of the 38 differentially expressed clones, seven genes were unknown, four melanoma-specific and three naevus-specific transcripts (Tables 2 and 3) including a sequence contained in a human BAC clone (RG180F08), four expressed sequence tags (ESTs), one unknown gene (clone 16 in Table 3) that shows a small homology to bovine desmoglein (81/92 bases), and one sequence that shows no homology to any known gene (clone 8 in Table 3). The latter revealed a very restrictive expression pattern, and it has yet to be clarified in which skin cell the gene is expressed. Although tissues from different patients were compared only two polymorphic genes were detected (HLA B and Ig $\lambda$).

Six of the identified genes were selected to further confirm differential gene expression on Northern blot including CAGH-3, dbpB/YB-1, EST ac73 g10.sl, 71-kDa heat shock cognate protein, the novel gene (clone 8 in Table 3), and 67-kDa laminin receptor. All six genes were confirmed to be differentially expressed between benign and malignant melanocytic lesions. The results of the array hybridizations were confirmed in five cases, reflecting the strength and usefulness of the technique. However, the sixth gene, the 67-kDa laminin receptor was found on Northern blot to be higher expressed in melanoma which is in line with published data (Vacca et al, 1993) but contradicts the finding of the array hybridization. This demonstrates that array hybridization is a good tool for screening large numbers of clones but Northern blot is necessary to confirm the results for the individual clones.

CAGH-3 was initially found by screening human brain cDNA libraries with $(CAG)_n$ probes to identify new candidate genes for neuropsychiatric diseases arising from trinucleotide repeat expansion mutations (Neri et al, 1996). Gene products with several glutamine residues may function as transcription factors and have a potential role in neurodevelopment (Margolis et al, 1997). The fact that CAGH-3 is also expressed in primary melanoma, melanoma metastases and melanoma cell lines (Figure 4C) could be explained by the origin of the melanocytes from the neural crest. CAGH-3 may be a transcription factor that is involved in the melanocytic transformation process. In addition, we found that

© 2000 Cancer Research Campaign 

CAGH-3 is expressed in high quantities in placenta, much higher than in brain and ovary, suggesting that CAGH-3 also plays a role outside the brain.

The 71-kDa heat shock protein shows an increase in gene expression with tumour progression. This gene is also expressed in normal melanocytes in vitro suggesting that there are substantial differences in the expression profile between benign melanocytic lesions and melanocytes in vitro (Figures 4F and 5C). This protein is supposed to have important roles as multifunctional chaperone including modulation of oncogene-mediated transformation, e.g. interaction with p53 (Yehiely and Oren, 1992).

dbpB/YB-1 is a member of a DNA-binding protein family, contains a cold shock domain and is regarded as transcriptional regulator (Kudo et al, 1995). Binding of dbpB/YB-1 to the epidermal growth factor receptor (EGFR) enhancer (Sakura et al, 1988) may lead to an up-regulation of EGFR. Interestingly, expression of EGFR correlates with melanoma progression (Rodeck, 1993). To our knowledge, expression of dbpB/YB-1 has never been associated until now with melanocytic transformation. In 27 out of 27 primary breast tumours dbpB/YB-1 was expressed but the gene product was undetectable in normal breast tissues (Bargou et al, 1997). The same group found that dbpB/YB-1 can induce MDR-1 gene expression and multidrug resistance in human breast cancer. We found dbpB/YB-1 in many tissues, mainly in skeletal muscle, testis and heart, confirming previous results (Kudo et al, 1995). In addition, dbpB/YB-1 is known to be a major, cell type-specific transactivator of matrix metalloproteinase-2 (MMP-2) transcription by mesangial cells (Mertens et al, 1997).

It is not known whether the genes identified here can be causally linked to melanocytic transformation but due to their known functions in other cells they are potential candidates for pathogenetic factors of melanoma. It remains to be demonstrated which of the identified genes are differentially expressed in melanocytes and which in the neighbouring other cell types, like keratinocytes or fibroblasts. The latter are also important for tumour progression (Herlyn and Shih, 1994) because growth stimulation of normal and malignant cells, cell motility, angiogenesis, stroma formation within malignant lesions and detachment of tumour cells from lesions involve complex interactions between autocrine, paracrine and endocrine factors (Herlyn and Malkowicz, 1991).

In conclusion, we have demonstrated that PCR-based subtractive hybridization in combination with high-throughput array hybridizations and conventional Northern blot is highly efficient to detect differentially expressed genes even in tissues from which high-quality RNA is difficult to isolate in sufficient amounts. Therefore, the approach should also be useful for other tumours to identify potential factors and novel genes involved in malignant transformation. The transcripts identified in this study represent valuable candidate genes for further functional analysis in malignant melanoma and should be informative in studying the biology of the tumour.

### ACKNOWLEDGEMENTS

The authors thank T Iftner and colleagues for DNA sequencing and M Schwarz and colleagues for the use of radioisotopes and Phosphorimager. The study was supported by a grant of the fortune programme of the Eberhard-Karls-University in Tuebingen.

### REFERENCES

Altschul SF, Madden TL, Schaffer AA, Zhang J, Zhang Z, Miller W and Lipman DJ (1997) Gapped BLAST and PSI-BLAST: a new generation of protein database search programs. *Nucleic Acids Res* **25:** 3389–3402

Bargou RC, Jurchott K, Wagener C, Bergmann S, Metzner S, Bommert K, Mapara MY, Winzer KJ, Dietel M, Dorken B and Royer HD (1997) Nuclear localization and increased levels of transcription factor YB-1 in primary human breast cancers are associated with intrinsic MDR1 gene expression. *Nat Med* **3:** 447–450

Clark WH Jr, From L, Bernardino EA and Mihm MC (1969) The histogenesis and biologic behavior of primary human malignant melanomas of the skin. *Cancer Res* **29:** 705–727

Diatchenko L, Lau YF, Campbell AP, Chenchik A, Moqadam F, Huang B, Lukyanov S, Lukyanov K, Gurskaya N, Sverdlov ED and Siebert PD (1996) Suppression subtractive hybridisation: a method for generating differentially regulated or tissue-specific cDNA probes and libraries. *Proc Natl Acad Sci USA* **93:** 6025–6030

Feinberg AP and Vogelstein B (1983) A technique for radiolabeling DNA restriction endonuclease fragments to high specific activity. *Anal Biochem* **132:** 6–13

Herlyn M (1993) *Molecular and Cellular Biology of Melanoma*. CRC Press: Boca Raton, FL

Herlyn M and Malkowicz SB (1991) Regulatory pathways in tumor growth and invasion. *Lab Invest* **65:** 262–271

Herlyn M and Shih IM (1994) Interactions of melanocytes and melanoma cells with the microenvironment. *Pigment Cell Res* **7:** 81–88

Hipfel R, Garbe C and Schittek B (1998) RNA isolation from human skin tissues for colorimetric differential display. *J Biochem Bioph Meth* **18:** 131–135

Ikeda H, Lethe B, Lehmann F, van Baren N, Baurain JF, De Smet C, Chambost H, Vitale M, Moretta A, Boon T and Coulie PG (1997) Characterization of an antigen that is recognized on a melanoma showing partial HLA loss by CTL expressing an NK inhibitory receptor. *Immunity* **6:** 199–208

Kudo S, Mattei MG and Fukuda M (1995) Characterization of the gene for dbpA, a family member of the nucleic-acid-binding proteins containing a cold-shock domain. *Eur J Biochem* **231:** 72–82

Margolis RL, Abraham MR, Gatchell SB, Li SH, Kidwai AS, Breschel TS, Stine OC, Callahan C, McInnis MG and Ross CA (1997) cDNAs with long CAG trinucleotide repeats from human brain. *Hum Genet* **100:** 114–122

Mertens PR, Harendza S, Pollock AS and Lovett DH (1997) Glomerular mesangial cell-specific transactivation of matrix metalloproteinase 2 transcription is mediated by YB-1. *J Biol Chem* **272:** 22905–22912

Neri C, Albanese V, Lebre AS, Holbert S, Saada C, Bougueleret L, Meier Ewert S, Le Gall I, Millasseau P, Bui H, Giudicelli C, Massart C, Guillou S, Gervy P, Poullier E, Rigault P, Weissenbach J, Lennon G, Chumakov I, Dausset J, Lehrach H, Cohen D and Cann HM (1996) Survey of CAG/CTG repeats in human cDNAs representing new genes: candidates for inherited neurological disorders. *Hum Mol Genet* **5:** 1001–1009

Real FX, Rettig WJ, Chesa PG, Melamed MR, Old LJ and Mendelsohn J (1986) Expression of epidermal growth factor receptor in human cultured cells and tissues: relationship to cell lineage and stage of differentiation. *Cancer Res* **46:** 4726–4731

Reed JA, Loganzo F, Jr., Shea CR, Walker GJ, Flores JF, Glendening JM, Bogdany JK, Shiel MJ, Haluska FG, Fountain JW and Albino AP (1995) Loss of expression of the p16/cyclin-dependent kinase inhibitor 2 tumor suppressor gene in melanocytic lesions correlates with invasive stage of tumor progression. *Cancer Res* **55:** 2713–2718

Rodeck U (1993) Growth factor independence and growth regulatory pathways in human melanoma development. *Cancer Metastasis Rev* **12:** 219–226

Sakura H, Maekawa T, Imamoto F, Yasuda K and Ishii S (1988) Two human genes isolated by a novel method encode DNA-binding proteins containing a common region of homology. *Gene* **73:** 499–507

Shih IM, Elder DE, Hsu MY and Herlyn M (1994) Regulation of Mel-CAM/MUC18 expression on melanocytes of different stages of tumor progression by normal keratinocytes. *Am J Pathol* **145:** 837–845

Siebert PD, Chenchik A, Kellogg DE, Lukyanov KA and Lukyanov SA (1995) An improved PCR method for walking in uncloned genomic DNA. *Nucleic Acids Res* **23:** 1087–1088

Vacca A, Ribatti D, Roncali L, Lospalluti M, Serio G, Carrel S and Dammacco F (1993) Melanocyte tumor progression is associated with changes in angiogenesis and expression of the 67-kilodalton laminin receptor. *Cancer* **72:** 455–461

van der Bruggen P, Traversari C, Chomez P, Lurquin C, De Plaen E, van den Eynde B, Knuth A and Boon T (1991) A gene encoding an antigen recognized by cytolytic T lymphocytes on a human melanoma. *Science* **254:** 1643–1647

 *© 2000 Cancer Research Campaign*

Wagner SN, Wagner C, Schultewolter T and Goos M (1997) Analysis of Pmel17/gp100 expression in primary human tissue specimens: implications for melanoma immuno- and gene-therapy. *Cancer Immunol Immunother* **44:** 239–247

Wang Z and Brown DD (1991) A gene expression screen. *Proc Natl Acad Sci USA* **88:** 11505–11509

Yehiely F and Oren M (1992) The gene for the rat heat-shock cognate, hsc70, can suppress oncogene-mediated transformation. *Cell Growth Differ* **3:** 803–809

Zhang L, Zhou W, Velculescu VE, Kern SE, Hruban RH, Hamilton SR, Vogelstein B and Kinzler KW (1997) Gene expression profiles in normal and cancer cells. *Science* **276:** 1268–1272

*© 2000 Cancer Research Campaign*
